# Evaluating risk of burnout and psychological challenges among forensic odontologists: A pilot study

**DOI:** 10.1007/s12024-025-01169-3

**Published:** 2026-02-10

**Authors:** Nikolaos Angelakopoulos, Aldani Wijayanti Putri, Sakher Jaber Al-Qahtani, Rizky Merdietio Boedi, Julie Langan Martin

**Affiliations:** 1 Department of Orthodontics and Dentofacial Orthopedics, University of Bern, Freiburgstrasse 7, Bern, 3010 Switzerland; 2 Faculty of Psychology, Universitas Diponegoro, Semarang, Central Java Indonesia; 3 Department of Pediatric Dentistry and Orthodontics, Clinical Forensic Odontology Unit, College of Dentistry, King Saud University, Riyadh, Saudi Arabia; 4 Department of Dentistry, Faculty of Medicine, Universitas Diponegoro, Semarang, Central Java Indonesia; 5 School of Health and Wellbeing, University of Glasgow, Glasgow, UK

**Keywords:** Burnout, Disaster victim identification, Forensic odontology, Mental health, Occupational stress, Psychological challenges, PTSD

## Abstract

Forensic odontologists (FOs) are routinely exposed to emotionally intense, high-stakes environments such as disaster victim identification (DVI) missions and criminal investigations. Despite the psychological demands of their role, there is limited empirical research on the mental health and burnout risk among FOs. This pilot study aimed to explore burnout risk and psychological challenges among FOs through an international online survey, with particular attention to trauma exposure, coping strategies, and access to mental health resources. A cross-sectional survey was disseminated via professional FO networks. The questionnaire included selected items from the Maslach Burnout Inventory (MBI) and trauma-related symptoms items adapted from PCL-5 DSM-5-TR PTSD criteria, alongside an open-ended question. Quantitative data were analyzed using R, and qualitative responses were examined through thematic analysis. A total of 105 responses were analyzed. Most participants (69.5%) reported DVI experience, and 40% had over 20 years of practice. Moderate risk of burnout levels were observed, with lower scores in more experienced FOs. Notably, respondents with repeated DVI exposure reported lower risk of burnout but elevated trauma-related symptoms. Only 9.5% had sought psychological support. Psychological coping training correlated with reduced risk of burnout. Thematic analysis highlighted recurrent needs for peer support, access to psychological care, resilience training, institutional recognition, and personal coping methods.FOs face persistent psychological risks that may be inadequately addressed by current systems. This study underscores the need for trauma-informed resources, institutional support structures, and targeted interventions to safeguard FO mental health in high-risk occupational forensic investigations.

**Supplementary information:**

The online version contains supplementary material available at https://doi.org/10.1007/s12024-025-01169-3.

## Keypoints

- Burnout Risk and Psychological Distress Vary by Experience and Age: Younger and less experienced forensic odontologists reported higher burnout levels, while older and more experienced professionals demonstrated greater resilience and a stronger sense of purpose.
- DVI Mission Participation Increases Psychological Strain Despite Enhancing Sense of Purpose: Forensic odontologists involved in multiple Disaster Victim Identification operations showed higher psychological distress but also reported a greater sense of personal accomplishment.
- Training and Peer Support Mitigate Risk of Burnout: Formal coping training and access to psychological support significantly reduce burnout symptoms, highlighting the importance of structured resilience programs and peer networks for forensic odontologists’ mental well-being.

**Supplementary information:**

The online version contains supplementary material available at https://doi.org/10.1007/s12024-025-01169-3.

## Introduction

Forensic odontology (FO), a niche subspecialty of dentistry and forensic sciences, plays a critical role in the identification of human remains, the analysis of bitemark evidence, dental age estimation, and dental malpractice investigations [1, 2]. Recent pilot research investigating the global demographics of forensic odontologists (FOs) indicates that their engagement in casework is predominantly occasional in nature [3]. The study further revealed the diverse institutional affiliations of these professionals, who may operate within police or military agencies, government bodies, academic institutions, or serve as independent consultants in FO [3]. The majority of FOs maintain full-time roles as dental practitioners while contributing to forensic efforts on an on-call basis, often in collaboration with forensic institutes and specialized centers. Additionally, many are integrated into national Disaster Victim Identification (DVI) teams or are affiliated with private emergency response organizations.

FOs work in high-pressure, emotionally taxing environments, such as disaster zones, where they contribute to DVI operations [3, 4]. They are often exposed to traumatic events, including mass casualties and criminal cases [5]. The nature of this work places professionals in challenging environments where exposure to distressing evidence, high-stakes decision-making, and legal pressures are common [6]​. As a result, FOs may experience elevated levels of occupational stress, potentially leading to burnout and adverse psychological challenges [7]​​.

Burnout, a psychological syndrome characterized by emotional exhaustion, depersonalization, and reduced personal accomplishment [8], is well-documented in healthcare [9–12] and forensic sciences [13]. However, there remains a paucity of research focusing specifically on burnout and psychological well-being among FOs, despite their critical role in legal and humanitarian contexts [5, 14]. Studies on forensic practitioners in other disciplines suggest that workplace stress can impair decision-making, reduce job satisfaction, and increase turnover​ [7]. FOs, given their exposure to similar stressors, may be at risk of comparable occupational health challenges [6]..

To address this gap, this study sought to evaluate the risk of burnout and psychological well-being among FOs using a pilot online survey. By identifying key stressors, coping mechanisms, and mental health outcomes, this research sought to contribute to a better understanding of occupational well-being in FOs. The findings may inform future strategies for stress management, professional support systems, and mental health interventions tailored to FOs.

## Materials and methods

### Survey participants

This cross-sectional descriptive study used a self-administered online survey to investigate the prevalence of burnout, psychological challenges, and the availability of support systems for FOs. Eligible participants included in the study were qualified FOs actively engaged in clinical forensic activities worldwide. The online survey was disseminated through various communication channels, including closed professional FOs WhatsApp^®^ groups, personalized LinkedIn^®^ messages, and emails sent to professional associations via their respective presidents, requesting them to share the survey link with their members. Individuals without a professional background as FOs would be excluded from the study. Given the nature of the survey dissemination, which allowed for potential forwarding by recipients to third parties not related to the FOs field, responses would be carefully screened, and individuals without verifiable FOs expertise or with inconsistent answers were excluded.

On 6 February 2025, the survey link was first shared via INFOdont, a closed WhatsApp^®^ group comprising 114 members from the International Network for Forensic Odontology. Additionally, personalized LinkedIn^®^ messages to 30 individuals within their professional network, encouraging participation in the survey. A further 10 individualized messages were sent to researchers identified through ResearchGate^®^ using search queries relevant keywords to search for expertise, such as “forensic odontology”, and “forensic dentistry”. Participants were invited to answer all questions, with an estimated average completion time of around 13 min.

A systematic approach was taken to disseminate the survey through FO associations and scientific societies. Contact information for key individuals within these associations was obtained from the membership directory on the International Organization for Forensic Odonto-Stomatology (IOFOS) website (https://iofos.eu/member-of-iofos/*)*, as well as from the official websites of the respective associations and a recently published study that examined the demographics of forensic odontology [3]. Personalized email communications were sent to these representatives or designated contacts, requesting their assistance in sharing the survey link with their members.

### Survey questionnaire

The questionnaire was created using Microsoft Forms (https://forms.office.com/Pages/), comprising 27 questions designed to collect comprehensive data. The survey included a combination of multiple-choices and one open-ended question. The questions were designed to collect information about (1) Demographics (3 questions), (2) FO’s working experience (4 questions), (2) DVI experience (4 questions), (3) Risk of burnout assessment adapted from the Maslach Burnout Inventory (MBI) (3 questions), (4) Help-seeking behavior (10 questions), and (5) Other psychological challenges (2 questions) and (6) Consent to participate (1 question). The questions were framed in a selection of specific items that were informed by validated instruments, such as the MBI, which has been extensively used in occupational burnout research [15, 16]. A targeted item selection strategy of burnout-related items was employed rather than the full MBI scale using corrected total items consideration [17, 18]. To measure burnout-related experiences, three items were adapted to reflect the core dimensions of the Maslach Burnout Inventory (MBI): emotional exhaustion, depersonalization, and personal accomplishment. Emotional exhaustion was assessed with the item (“*How often do you feel physically and emotionally exhausted at the end of your workday?”* (Item 17)), which showed a high factor loading (0.79) on the burnout factor in prior research [19]. Depersonalization was assessed with one item (“*How often do you feel emotionally detached or less empathetic toward others as a result of your work in forensic odontology?”* (Item 16)), which loaded at 0.80 on its factor [19]. To represent personal accomplishment, participants responded to the statement (“*Despite its psychological challenges*,* this role offers a sense of purpose”* (Item 26)), item loaded 0.75 on its intended factor, indicating a strong association [19]. This decision follows methodological precedents where abbreviated versions of validated scales have been effectively utilized to maintain construct validity while reducing participant burden in exploratory pilot designs [20, 21].

Additionally, studies have supported the use of shortened versions of the Post traumatic Stress Disorder Checklist for DSM-5 (PCL-5)—a self-report screening tool based on the Diagnostic and Statistical Manual of Mental Disorders, Fifth Edition (DSM-5). These brief versions retain the core symptom clusters defined by the DSM-5, while reducing the number of items to ease the burden of assessment in occupational settings [22, 23]. In this study, two items (Item 13 and Item 14) were developed to reflect psychological challenges frequently encountered in forensic contexts. While informed by the core symptom domains of the PCL-5 (such as intrusion, arousal, and negative alterations in cognition). These items were not intended to constitute a formal PTSD diagnostic assessment. Item 13 captured the perceived psychological toll of forensic work, encompassing emotional exhaustion, vicarious trauma, depressive and anxious states, chronic occupational stress, and difficulties with work-life balance. Item 14 focused on trauma-related somatic and cognitive disruptions, including flashbacks, intrusive imagery, hypervigilance, concentration problems, sleep disturbances, and somatic health complaints. Both items were developed with input from JLM, a psychiatrist and academic with expertise in mental health. They were reviewed through expert judgment to map key psychological stress responses associated with forensic work exposure, while avoiding overdiagnosis or strict adherence to clinical PTSD criteria [24].This study integrates burnout assessment with psychological stressors and help-seeking behavior, aligning with contemporary disaster response and forensic psychology research [16, 25]. The combination of burnout measures with trauma exposure and coping strategies enhances the understanding of psychological well-being in high-risk professions [26, 27]. A mixed-methods approach, incorporating Likert-scale and open-ended questions, facilitates both statistical analysis and thematic exploration of coping mechanisms and professional support needs. While primarily quantitative, the inclusion of open-ended responses aligns with the growing recognition of qualitative survey methods in applied psychology research [28]. These responses allow for participant-driven insights and the identification of actionable recommendations. To ensure validity and clarity, survey items were reviewed by two experts in psychometry and psychology, with refinements made to improve measurement accuracy and conceptual relevance for FOs. The experts assessed measurement accuracy and response scaling, while the psychology expert reviewed conceptual relevance and readability. Their feedback was incorporated to refine the wording and improve the survey’s precision and applicability to FOs.

### Ethical considerations

All participants were provided with detailed information about the research objectives and procedures before participating in the study. Informed consent was obtained electronically prior to completing the questionnaire. All data were collected anonymously and stored securely, ensuring that participants’ personal information was protected. Any data that could potentially reveal their identity were handled with the utmost confidentiality. Participation in the study was entirely voluntary, and participants were free to withdraw at any stage without facing any consequences or needing to provide a reason. The outlined investigation protocol received approval from the Ethics Committee of Faculty of Medicine Universitas Diponegoro, Indonesia (approval number: 092/EC/KEPK/FK-UNDIP/IV/2025).

### Analysis

All data analyses were done using R (version 4.3.1, R Foundation for Statistical Computing, Vienna, Austria). The questions taken from the Maslach Burnout Inventory (i.e., Q15, Q16, Q20) were taken as a score from Likert 0 (never) to 5 (always) and converted to a mean value (sum of score/3). Basic descriptive statistics were used to summarize the demographics. Additionally, various data from the demographics were analyzed quantitatively for each primary variable using appropriate quantitative analysis. For example, t-test analyses were undertaken to observe the difference between DVI involvement (yes/no) to MBI score, or Multiple Regression and ANCOVA to analyze the changes and moderating variables.

In addition to collecting demographic data, open-ended responses at the end of the questionnaire were analyzed thematically using Braun and Clarke (2006) approach [28]. Responses were coded, grouped into “meaning units”, and categorized under consolidated codes. Similarities and differences were examined, and through team discussions, “overarching themes” were developed.

## Results

The survey recorded 106 responses; however, one response was excluded due to an inconsistency (i.e., DVI involvement was answered as “No” while the number of DVI involvements was reported as “3–5”), resulting in a final sample of 105 responses. Most respondents were between 45 and 54 years of age (*n* = 25, 23.8%) and female (*n* = 60, 57.1%). The majority were highly experienced, with more than 20 years of practice (*n* = 42, 40%) (Table 1).

**Table 1:** Demographic characteristics of survey respondents

Respondents		*n*	Proportion
Demographics			
Age	18–24	0	0
	25–34	10	9.5%
	35–44	17	16.2%
	45–54	25	23.8%
	55–64	24	22.9%
	over 65 years old	29	27.6%
Sex	Man	45	42.9%
	Woman	60	57.1%
Forensic Odontology Experience			
Years of Experience	< 5	16	15.2%
	5–10	19	15.2%
	11–20	26	24.8%
	> 20	42	40%
Working Areas	Bitemark Analysis	30	14.6%
	Dental Age Estimation	60	29.3%
	Dental Malpractice Cases	24	11.7%
	Dental Identification	91	44.4%
Participation in DVI	Yes	73	69.5%
	No	32	30.5%
Number of DVI participation	1–2	28	26.7%
	3–5	19	18.1%
	> 5	26	24.8%
	None	32	30.5%
Demographics	Africa	2	1.9%
	Asia	24	22.6%
	Europe	26	24.5%
	North America	20	18.9%
	Oceania	22	20.8%
	South America	11	10.4%
	Not Reported	1	0.9%

Most respondents participated in DVI operations (*n* = 73, 69.5%), with 26 having participated in more than five operations. This finding is reflected in the areas of work reported: the majority were involved in dental identification cases (*n* = 91, 44.4%), whereas dental malpractice cases were less common (*n* = 24, 11.7%).

Table 2 presents average MBI scores for each respondent characteristic. An initial reliability analysis of the survey, conducted with 40 participants, gives Cronbach’s alpha of 0.69. Although the overall MBI score did not significantly differ across demographic groups, the “sense of purpose” item that was used to assess the MBI score diferred between age groups and DVI involvement, with those who had participated in DVI operations reported a significant change in “sense of purpose” scores (β = 0.48 95% CI [0.07, 0.89], F = 5.3, f*p* < 0.05) with a medium effect size (d = 0.49). Specifically, FOs in older age groups reported a “sense of purpose” for the job approximately 1 point higher than their younger counterparts, reflecting a greater sense of personal accomplishment, one of the core dimensions assessed in MBI.

The analysis of PCL-5 scores in relation to respondents’ DVI mission experience (Table 2) gives no significant differences between categories. However, further examination of the PCL-5 components using t-test initially indicated that FOs who participated in DVI operations reported a significantly higher number of psychological symptoms than those who had never participated. However, when subjected to linear regression model, the association did not reach statistical significance (β = 0.39, 95% CI [−0.03, 0.8], F = 3.4, *p* = 0.067). This difference was not observed on the number of DVI operations.

**Table 2:** Maslach burnout inventory score and PTSD checklist for DSM-5 score observed in forensic odontologists

Respondents		Risk of Burnout Score		PCL-5 Score	
		Mean	S.D	Mean	S.D
Age	25–34	7.2	1.81	1.2	1.62
	35–44	6.5	1.62	1.65	1.97
	45–54	6.6	1.82	1.92	2.02
	55–64	6	1.76	1.38	2.12
	over 65 years old	6.72	1.86	0.828	1.17
Sex	Man	6.17	1.93	1.56	1.8
	Woman	6.35	1.74	1.25	1.83
Participation in DVI	Yes	6.27	1.98	1.52	1.90
	No	6.28	1.75	1.06	1.58
Number of DVI participation	1–2	6.17	1.82	1.46	2
	3–5	6.94	1.95	1.42	1.35
	> 5	5.88	1.42	1.65	2.17

**Fig. 1:**
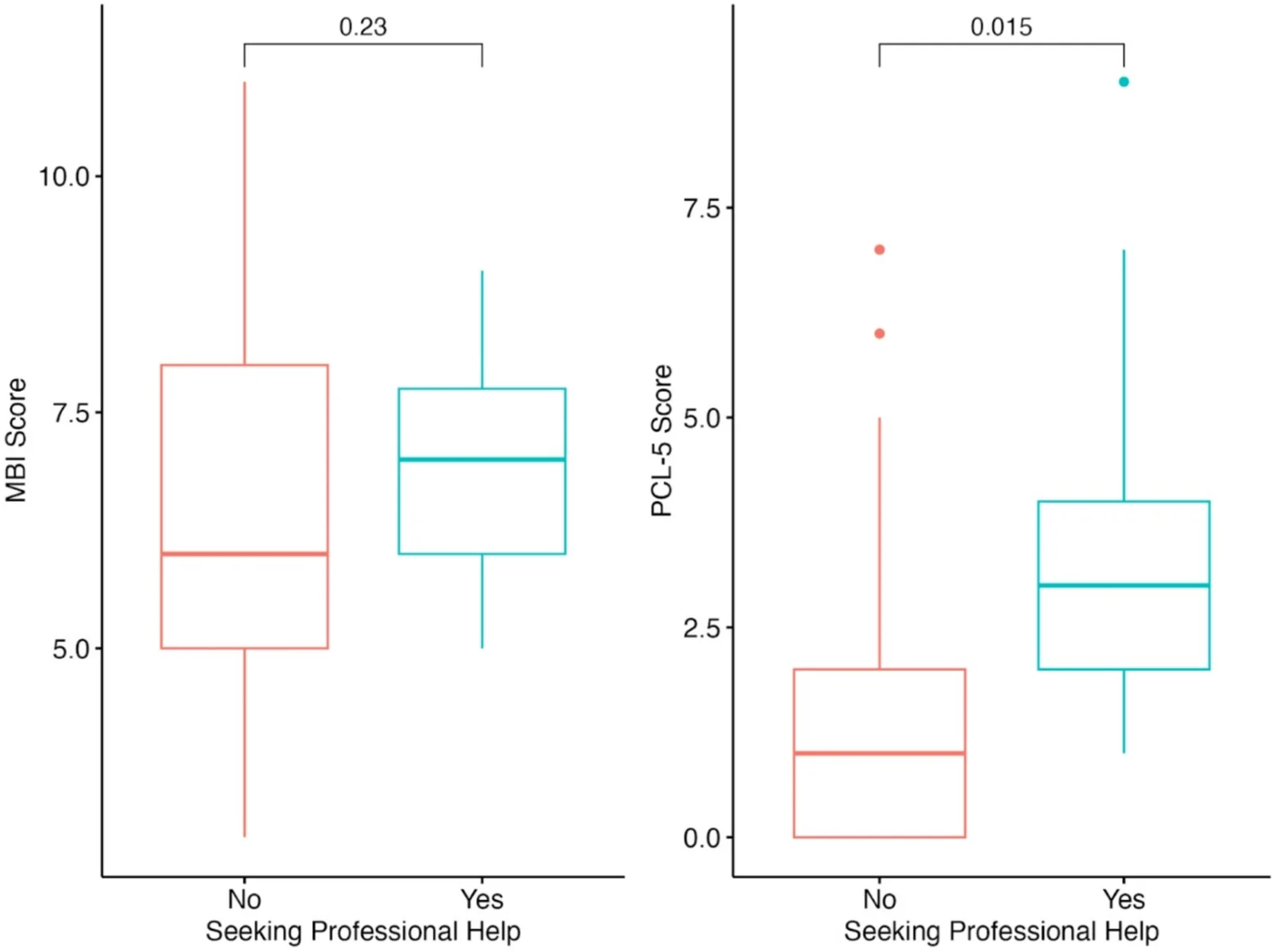
Differences in MBI and PCL-5 score related to help seeking behaviour of a forensic odontologist

The majority of respondents (*n* = 95) have not pursued professional help related to their FOs work, and significant differences were observed in the PCL-5 scores (Fig. 1). Specifically, FOs whom seeking professional help has a higher PCL-5 scores (β = 1.35, 95% CI [0.74, 1.96], F = 19.44) with an R^2^ of 0.16 and large effect size (*d* = −1.47).

Forty respondents indicated that they received adequate training to cope with the psychological challenges associated with their professional work, 11 reported receiving some training that was insufficient, and 54 reported receiving no training.

**Fig. 2:**
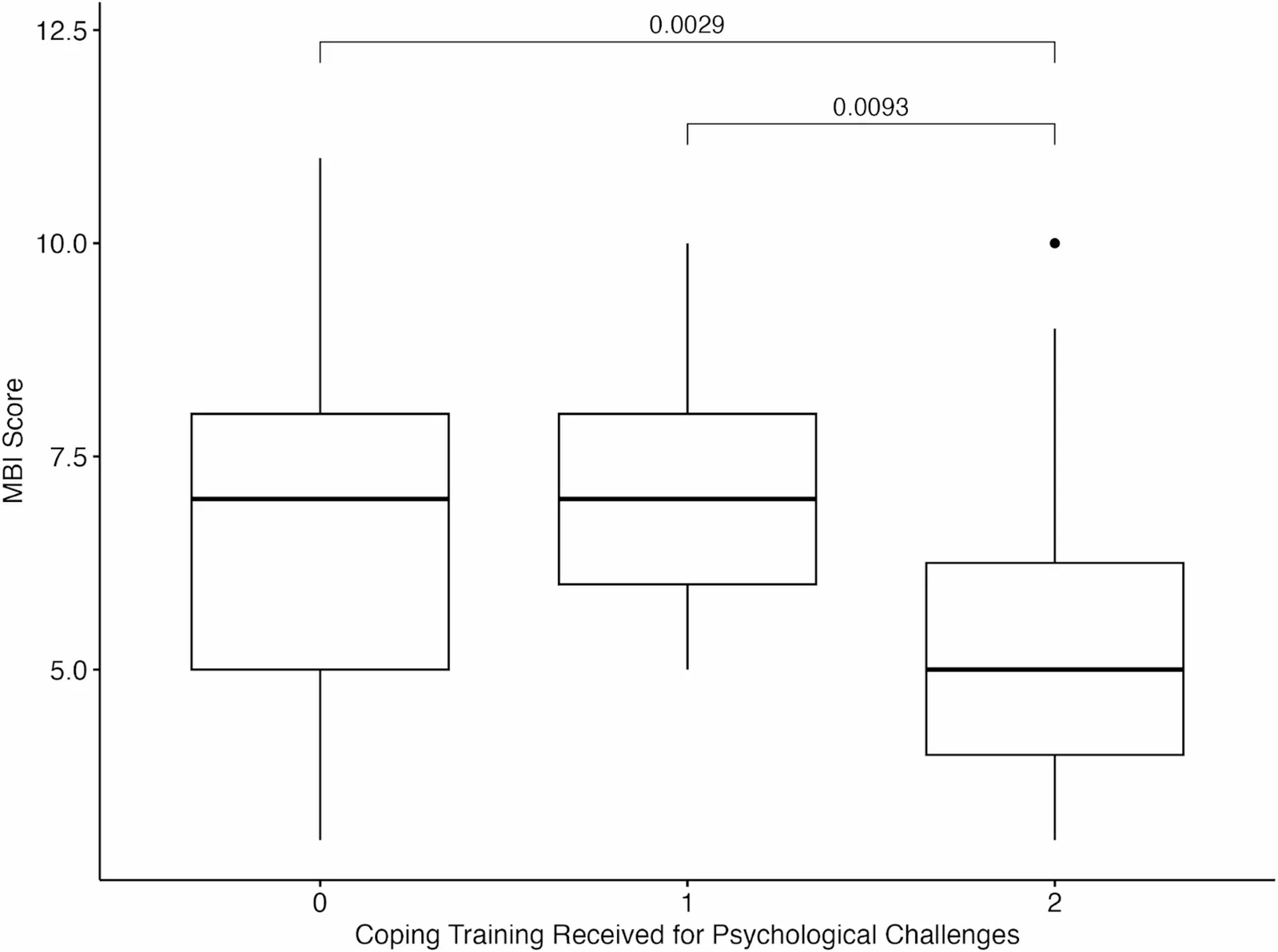
MBI scores differences in coping training received by forensic odontologist. 2 = Received sufficient training, 1 = Received training but not sufficient, 0 = Did not receive training or Not sure

These differences in training were reflected in the MBI scores. Further analysis confirmed a significantly lower MBI scores compared with insufficient or no training (Estimated = −1.10, *p* < 0.05) with a negative association (β = − 0.61, 95% CI [−1, −0.21]) with an R^2^ of 0.12. However, no significant difference was observed in the PCL-5 scores (Fig. 2). Additionally, a significant positive correlation between MBI and PCL-5 score was observed (*r* = 0.35, 95% CI [0.17, 0.5], *p* < 0.05). Further linear model analysis gives PCL-5 a positive association with MBI score (β = 0.35, 95% CI [0.17, 0.53], F = 14.27, R^2^ = 0.11). It should be noted that ANCOVA analysis did not give any significant moderating variables of all the analysis above.

While the quantitative results provided an overview of burnout risk and psychological strain across participants, they could not fully capture the coping strategies and unmet support needs expressed by forensic odontologists. To deepen our understanding of these complexities, respondents were invited to elaborate on their experiences through an open-ended question. This qualitative input offers valuable context, revealing themes not readily apparent in scaled responses. The following thematic summary illustrates the psychosocial challenges and highlights what resources would meaningfully improve well-being as an FOs using Braun and Clarke (2006)’s thematic analytic approach [28].

Thematic analysis from open-ended questionnaire yielded five overarching themes:

- Peer support and collegial networks, in which participants emphasized the need for consistent opportunities to connect with colleagues and share experiences as a foundational need. One respondent wrote, “*Discussions of peer groups to share experiences and feelings”* [R23], while another suggested, “*Regularly ventilation talking with colleagues*,” [R40] highlighting the role of collegial contact in promoting emotional resilience. One noted, “*The work itself is rewarding. Lack of opportunities to participate*,* exclusion by colleagues*,* was very stressful. Threats and intimidation tactics from colleagues have been surprising to encounter. Forensics is a demanding field and requires a collaborative environment to accomplish our tasks well. I am thankful for the colleague who have provided opportunities and mentorship*,* respect and acceptance. A collegial environment is key in managing the stresses and quality of our work*” [R22]. These sentiments reflect a perceived need for reflective dialogue and mutual validation.
- Psychological support and counselling reflected a widespread recognition of the need for formalized mental health infrastructure within FOs practice. “*Dependent on the situation. Single recovery can be just as emotionally draining as large accidents”*[R35]. Several participants underscored the necessity of trauma-informed care in operational procedures. One respondent proposed, “*Psychologists to be part of DVI teams and mandatory debriefing after each mission”*[R17]. Respondent suggest that psychological recovery should be institutionalized, not incidental. Others emphasized integrated wellbeing measures, such as “*Peer support groups*,* good debriefing*,* meditation/breathwork together”* [R18] pointing to the value of both clinical and community-based strategies.
- Training and education emerged as a critical theme, reflecting a widespread concern that many FOs are not adequately prepared for the psychological demands of their role. Participants highlighted the need for structured resilience training, regular psychological assessments, and curricula that go beyond technical instruction. One participant stated, “*Forensic odontologists have to be made aware of the possible psychological consequences of the job they are exercising. It would be recommended for them to receive specific training in psychology and the effects of the work they are doing.”* [R18] Another respondent reinforced this by suggesting, “*Identification of the impact of our work to our well-being*,* relevant courses and guidance during our FOs training*,* semestrial follow-up and psychological assessment within DVI teams.”* [R1]. These comments underscore the perceived inadequacy of current training models, which often focus on technical competence while overlooking the psychological toll of forensic work.
- Organizational and institutional support which highlighted the respondent’s calls for systemic changes to improve professional resources, encompassed access to psychologists or social workers, guaranteed breaks, structured debriefing, and reduced administrative burdens. “*Enough salary*,* guaranteed breaks*,* reduce administrative work*,*”* [R41]. Another respondent adds, “*Decent salary*,* long-term job stability*,* support from senior managers*,*”* [R11].
- Work-life balance and self-care strategies emerged as a theme reflecting the diverse ways FOs manage occupational stress outside institutional frameworks. Respondents described using activities such as “*physical exercise in combination with meditation*,*”*[R4] and “*meditation awareness*,* personal/professional balance*,* physical activity.”*[R21]. Other mentioned “*Hobbies or leisure activities”* [R7] as ways to manage stress. These responses reflect a range of self-care strategies used outside formal support systems.

## Discussion

To our knowledge, this is the first study that focused on FOs risk of burnout and psychological challenges, particularly those who previously worked in DVI missions. As a pilot investigation employing abbreviated screening measures, this study provides exploratory evidence regarding burnout risk among FOs. General physical and mental health are essential for avoiding professional burnout. Recent evidence on burnout and PTSD among forensic medicine workers indicates that these issues are caused by various factors, including job satisfaction, continuous exposure to autopsies, working with victims’ families [29] as well as age and support from co-workers and spouses [30]. A recent study [6] noted that although FO is a niche subspecialty of dentistry, the working environment for FOs differs significantly from that of general dental practice. For example, dentists rarely face death-related events, whether through witnessing death firsthand or engaging with grieving families. In this research, we explore the various factors that may have affected burnout and other psychological distress among FOs globally.

Burnout and psychological distress were found to vary across demographic age groups. Although the differences were not statistically significant, it was noted that the younger age group had a higher average of MBI, whereas the older age group exhibited moderate levels. Similar findings were confirmed by suggesting that psychological resilience tends to increase with experience and age [30, 31]. Even so, these higher scores in the younger age group can be mitigated by providing adequate training in post-graduate FOs curriculum or other training programs, such as DVI simulation.

The analysis also reveals gender-based differences in risk of burnout and psychological distress. Although the differences are relatively minor, men exhibit an MBI score of 6.17, while women have a slightly higher score of 6.35, indicating a marginally greater tendency toward burnout. Similar research by Almazrouei et al., (2021) found that female forensic examiners reported higher stress levels than males [7]. Sheshah et al., (2021) also found that female gender as a significant predictor of high burnout among Egyptian forensic physicians [32]. However, elevated trauma-related symptoms scores appear to be higher among men (1.56) than women (1.25), implying that male respondents may experience greater psychological distress. This is less commonly reported and may reflect gender-based differences in coping mechanisms used in response to occupational stress.

Participation in DVI missions appears to have a significant impact on psychological health. The data indicates that professionals engaged in more than five DVI missions exhibit the lowest MBI scores (5.88) but simultaneously record the highest PTSD scores (1.65). Exposure to trauma in general was reported by Shigemura et al., (2018) that various factors other than identifying bodies, such as property loss, anxiety for the future, can also bring a higher distress to dentists deployed in DVI operations [33]. This suggests that, while frequent DVI participation may enhance professional coping strategies, prolonged exposure to trauma could contribute to cumulative psychological distress from various factors. Given these findings, it is crucial to investigate strategies that mitigate the long-term psychological effects of such work and support for mental health should be provided in every DVI missions.

The importance of stress management and training in DVI missions was an important factor to ensure lower burnout and distress. Even so, Goldstein et al., (2021) found that mental health issues were insufficiently addressed during training [34]. In this research, respondents who received sufficient coping training display significantly lower MBI scores with mean MBI value of 5.52 ± 1.72 compared to those who received insufficient or no training, with MBI value of 7.27 ± 1.74 and 6.63 ± 1.73, respectively. Furthermore, access to psychological support and post-mission debriefing appears to enhance professional well-being, as evidenced by a 0.5-point increase in sense of purpose among trained FOs. As such, integration of both stress management training in the educational curriculum and in DVI sessions is mandatory to ensure the psychological resilience of FOs.

Burnout levels can vary depending on career stage. It was found that FOs with less than five years of experience have a higher MBI score of 7.2, which is significantly different from professionals with over 20 years of experience (MBI = 5.88). Similarly, Sebastian et al., (2023) found that experienced FOs has a lower discomfort score when dealing with child abuse cases, whereas trainees showed more stress and discomforts [5]. Various studies have identified similar trends in psychological resilience, demonstrating that coping tends to improve with age and professional experience—likely due to greater exposure, enhanced emotional regulation, and the development of adaptive coping strategies over time [7, 35, 36]. These findings suggest that targeted interventions for early-career professionals may be beneficial in reducing burnout rates.

Existing studies have highlighted the significant impact of occupational burnout among frontline disaster responders, emphasizing its correlation with high psychological stress, workload, and exposure to traumatic events [37, 38]. Prior research has noted the importance of coping mechanisms, such as peer support, psychological counseling, and structured debriefing sessions, in mitigating burnout-related symptoms [39]. However, despite the literature on burnout interventions, findings remain inconsistent regarding the most effective coping strategies, particularly in forensic professionals and other specialized disaster-response workers [40]. The inverse association between DVI experience and burnout, alongside elevated trauma-related symptoms, may reflect two concurrent psychological processes, namely habituation and cumulative trauma. Repeated exposure to disaster settings may lead to a degree of emotional habituation, whereby FOs become increasingly familiar with operational demands, develop task-focused coping strategies, and experience reduced emotional exhaustion and depersonalization over time. Such adaptive desensitization has been described among disaster responders and emergency professionals, where repeated exposure can attenuate burnout-related responses without eliminating emotional impact [41, 42]. At the same time, habituation does not preclude cumulative trauma. Each DVI deployment may contribute incrementally to the overall psychological burden, particularly through repeated exposure to human remains, grieving families, and high-stakes identification work. This accumulation of traumatic material may manifest as persistent trauma-related symptoms, even among highly experienced professionals who report lower risk of burnout. Similar patterns have been observed in disaster and humanitarian workers, where operational competence and role mastery coexist with long-term psychological strain (Benedek et al., 2007; Brooks et al., 2015) [43, 44]. These findings suggest that reduced burnout among experienced FOs may reflect adaptive occupational functioning, while elevated trauma symptoms indicate an underlying cumulative psychological cost of repeated DVI exposure.

The themes emerge from the last section of the generated questionnaire found that peer support and mentorship programs played a crucial role in reducing burnout. This finding is aligned with existing research that highlights the protective function of collegial networks and shared experiences [45]. Another finding is that psychological support services, including therapy and structured mental health check-ins, were frequently mentioned as essential resources, a conclusion that is consistent with previous studies demonstrating the benefits of early intervention and routine psychological evaluations [46].

Interestingly, while the current study identified work-life balance strategies, such as meditation and scheduled recovery time, as effective coping mechanisms, this aspect has been less emphasized in prior research, which has predominantly focused on institutional and policy-driven solutions [47]. A possible explanation for this discrepancy is that disaster responders, particularly those in high-risk forensic roles, may turn to faith as a means of finding meaning in their work, which aligns with research on existential coping in high-stress professions [45]. The finding that DVI participation increases both trauma symptoms and sense of purpose fits with models of post-traumatic growth and vocational calling. Park’s (2010) meaning-making framework suggests that confronting a severe stressor can trigger cognitive efforts to restore meaning, which in turn preserves one’s sense of purpose [48]. In this case, working on DVI tasks may reinforce a practitioner’s perception that they are ‘making a difference,’ thereby elevating their personal sense of accomplishment. Likewise, research on vocational calling shows that finding one’s work deeply meaningful buffers stress: physicians with higher awareness of meaning report substantially lower burnout and fatigue, and forensic professionals with higher life meaning have significantly lower burnout. Schnell’s work similarly underscores that meaningfulness protects against distress [49]. Together, these theories help explain our paradoxical outcome: engaging in demanding, trauma-exposed work can simultaneously heighten distress and enhance the practitioner’s sense of purpose.

Furthermore, while previous studies have reported administrative burden and lack of professional recognition as major contributors to burnout [38], this study found that many respondents emphasized the importance of peer validation and acknowledgment from colleagues over institutional recognition. This finding suggests that social and emotional support within professional networks may act as a crucial buffer against stress, independent of formal organizational policies.

Several limitations must be acknowledged. First, to minimize participant burden, we employed abbreviated measures rather than full diagnostic scales. The use of three MBI items, while enabling feasibility, captures only partial dimensions of the full burnout construct (emotional exhaustion, depersonalization, and personal accomplishment). While these items demonstrated high factor loading and expert validation, the internal consistency (Cronbach’s alpha = 0.69) suggests findings should be interpreted as exploratory risk indicators rather than clinical diagnoses. Second, recruitment via digital networks introduced selection bias, skewing the sample toward engaged, experienced practitioners (40% having > 20 years of experience). Additionally, the final sample size (*n* = 105), while adequate for a pilot analysis, limits the extent to which findings can be generalized to the global forensic community. Consequently, these findings may not fully reflect the experiences of isolated FOs and may overestimate the availability of peer support. Future research should prioritize larger sample sizes and more diverse recruitment strategies, including offline participation. Longitudinal studies are also recommended to monitor the progression of burnout and psychological distress over time. Tracking changes across different career stages and levels of exposure would allow researchers to more accurately identify key risk factors, protective mechanisms, and critical intervention points to help mitigate burnout among FOs. This study serves as a case example of how forensic professionals can experience both heightened trauma-related symptoms and a strengthened sense of professional purpose. As such, it may inform broader discussions on occupational health support across forensic domains.

## Conclusion

This study provides important insights into the psychological well-being and risk of burnout among FOs, particularly those involved in DVI missions. Our exploratory findings suggest several important results. Preliminary evidence indicates that FOs experience moderate levels of burnout-related symptoms, with younger and less experienced professionals appearing most vulnerable. This survey suggests that professional experience may buffer against risk of burnout, yet prolonged trauma exposure may still contribute to cumulative psychological strain. While participation in DVI missions appears to enhance a sense of purpose, it is also associated with increased psychological distress, highlighting the need for mental health support in this high-risk profession.

The study also emphasizes the protective role of coping training, peer support, and psychological interventions in mitigating burnout and stress-related symptoms. Participants who had received formal coping training or psychological services exhibited elevated distress levels. These patterns indicate that supports may serve as buffers against occupational burnout. The consistency of thematic findings with quantitative patterns strengthens confidence in the overall narrative.

Given the critical role FOs play in legal and humanitarian contexts, addressing their mental health needs should be a priority. Institutions and professional organizations should implement structured psychological resilience training, encourage peer support networks, and provide accessible mental health services to reduce occupational stress. Future research should explore longitudinal trends in burnout and psychological well-being, using larger and more diverse samples to develop targeted interventions that promote long-term occupational health in FOs. Most importantly, the present evidence provides a sufficient preliminary basis for developing targeted, trauma-informed support systems for practitioners, particularly those involved in DVI. Moreover, intervention studies are needed to empirically evaluate the effectiveness of various coping strategies, training, and debriefing in mitigating psychological distress in high-risk FOs roles.

## Supplementary information

Below is the link to the electronic supplementary material.

Supplementary File 1 (DOCX 9.17 KB)

## Data Availability

The data supporting this study’s findings are available on request from the corresponding author.
