# Supplementary material for: Evaluating risk of burnout and psychological challenges among forensic odontologists: A pilot study
Source: Forensic Sci Med Pathol. 2026 Feb 10;22(2):725–36. doi: 10.1007/s12024-025-01169-3 (PMC13652889; doi:10.1007/s12024-025-01169-3)
Supplement: Supplementary file 1 [file 12024_2025_1169_MOESM1_ESM.docx]

**Table 3.** Summary of Themes Identified from Open-Ended Question Responses

| **Overarching themes** | **Subthemes** |
| --- | --- |
| Peer Support and Collegial Networks | Peer support groups, peer group chats, and meetings |
|  | Regular discussions and debriefings with colleagues |
|  | Collegial validation |
| Psychological Support and Counseling | Regular psychological check-ups, mental health evaluations, and post-deployment debriefing |
|  | Access to professional psychological support (psychologists, therapists, or social workers) |
|  | Psychological support groups specifically for forensic odontologists |
| Training and Education | Courses, workshops, and programs on psychological resilience and stress management |
|  | Open forums to discuss forensic cases and psychological stress |
|  | Inclusion of psychological talk and coping strategies in forensic odontology training |
| Organizational and Institutional Support | Mentorship programs for younger forensic odontologists |
|  | More professional recognition from legal and law enforcement bodies |
|  | Better salaries, guaranteed breaks, and reduced administrative work |
| Work-Life Balance and Self-Care Strategies | Awareness of self-care techniques and early identification of stress symptoms |
|  | Relaxation (meditation, mindfulness, breathwork), and Hobbies (physical exercise, etc) |
|  | Encouraging activities that provide emotional relief, such as storytelling, gathering with loved ones, and sharing experiences |
